# Hyperbaric oxygen therapy sensitizes nimustine treatment for glioma in mice

**DOI:** 10.1002/cam4.851

**Published:** 2016-10-13

**Authors:** Zhaohui Lu, Jiawei Ma, Bing Liu, Chungang Dai, Tao Xie, Xiaoyu Ma, Ming Li, Jun Dong, Qing Lan, Qiang Huang

**Affiliations:** ^1^ Department of NeurosurgeryThe Second Affiliated Hospital of Soochow University1055 Sanxiang RdSuzhou215004China; ^2^ The Experimental CenterThe Second Affiliated Hospital of Soochow University1055 Sanxiang RdSuzhou215004China

**Keywords:** Chemotherapy, glioma stem cell, green fluorescence transgenic nude mice, hyperbaric oxygen therapy, immunity inflammation, transplantation model

## Abstract

Nimustine (ACNU) has antitumor activities in patients with malignant glioma. Hyperbaric oxygen (HBO) may enhance the efficacy of certain therapies that are hampered by the hypoxic microenvironment. We examined the combined effects of ACNU and HBO in a GFP transgenic nude mice bearing human glioma model. Mice inoculated with human glioma cells SU3 were randomly divided into the four groups: (A) the control group, (B) the HBOT (HBO therapy) group, (C) the ACNU group, and (D) the HBOT+ACNU group. Tumor size was measured at the indicated time intervals with a caliper; mice were sacrificed 28 days after treatment, and immunohistochemistry staining and western blot analysis were carried out. By the end of the trial, the tumor weights of groups A, B, C, and D were (*P *< 0.05), 6.03 ± 1.47, 4.13 ± 1.82 (*P* < 0.05), 2.39 ± 0.25 (*P* < 0.05), and 1.43 ± 0.38 (*P* < 0.01), respectively. The expressions of TNF‐*α*, MMP9, HIF‐*α*, VEGF, NF‐*κ*B, and IL‐1*β* were associated with the infiltration of inflammatory cells and the inhibition rate of tumor cells. Hyperbaric oxygen therapy (HBOT) could inhibit glioma cell proliferation and inflammatory cell infiltration, and exert a sensitizing effect on ACNU therapy partially through enhancing oxygen pressure (PO_2_) in tumor tissues and lower expression levels of HIF‐1*α*, TNF‐*α*, IL‐1*β*, VEGF, MMP9, and NF‐*κ*B.

## Introduction

Hyperbaric oxygen therapy, the noninvasion treatment, has been widely used in many common diseases, such as carbon monoxide poisoning. Almost all the hypoxic diseases were adaptive 1, however, there had been debates over HBO therapy for cancer patients in the past few decades 2, 3. By using the HBO therapy in mice bearing tumor, Stuhr et al. 4 proposed that hyperbaric oxygen might inhibit the growth of glioma cells, subcutaneously transplanted in C57BL/6J mice. It had been reported that HBOT combined with 5‐FU and doxorubicin had increased sensitivity in the treatment of solid tumors in mice 5, 6. However, HBOT did accelerate tumor's growth 7. And it was showed that HBOT promoted the pulmonary metastasis of breast cancer of C3H mouse 8.

The reasons for the debate were difficult to distinguish. It was believed that the HBO therapy efficacy might be tumor cell‐type specific. In this report, the glioma‐bearing animals were treated with HBOT, ACNU, or the combination of both. The whole experiment was carried in the fully closed and individual ventilated cages (IVC), which guaranteed that the nude mice lived in SPF environment, and were not affected by HBOT. We found that the hyperbaric oxygen therapy significantly increased the sensitivity of nimustine treatment for mice bearing glioma.

## Material and Methods

### Mice and reagents

**Figure 1:**
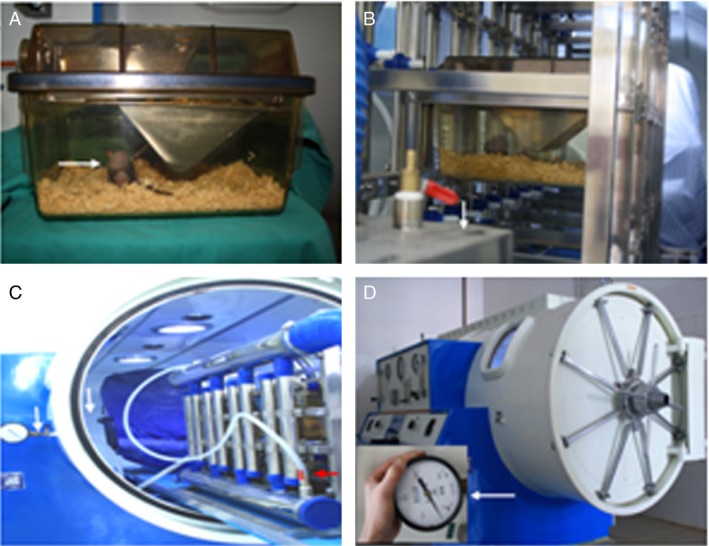
Hyperbaric oxygen—individual‐ventilated cage (HBO‐IVC) system. (A) A tumor‐bearing mouse (arrow) inside the IVC; (B) fully enclosed transport channel and the air filter apparatus (arrow) connecting the feeding room to the hyperbaric oxygen therapy (HBOT) room; (C) standby HBO‐IVC with plastic hose connecting the exhaust manifold (red arrow) and the pressure valve (white arrow) on the window; (D) working HBO‐IVC with manometer monitoring the pressure inside IVC. The mice were divided into the four groups randomly, and each group had five mice, as shown in the Material and Methods section.

The human glioma stem/progenitor cell line SU3 and nude mice expressing EGFP (NC‐CB57/6J‐EGFP) were prepared by our laboratory 9, 10. The NC‐C57BL6J‐EGFP mice (6–8 weeks of age) were established by our group and maintained in independent ventilation cages. The independent ventilated cage barrier system used in the feeding room and in the hyperbaric oxygen cabin was developed jointly with Suzhou Suhang Science & Technology Co., Ltd. (Jiangsu China) (Fig. 1). Hyperbaric oxygen chamber was purchased from Ningbo Medical Hyperbaic Oxygen Chamber Factory (Zhejiang China). Nimustine was purchased from Daiichi Sankyo Company Ltd. (Beijing China). Fluorescence microscope was purchased from Carl Zeiss (Shanghai China). HIF‐*α*, VEGF, MMP9, NF‐*κ*B, and TNF‐*α* antibodies were purchased from Abcam. (USA). IL‐1*β* antibody was purchased from Cell Signaling Technology (USA).

### Mouse model

The mice used in this study were aged 6–8 weeks and had a body weight of ~22 g. All the mice were bred and maintained in the specific pathogen‐free animal care facility. SU3 cells (5 × 10^6^ cells in 80 *μ*L of PBS) were subcutaneously injected in the flank of the nude mice. The mice were divided into the four groups randomly: (A) control group (untreated), (B) HBOT (only), (C) ACNU (only), (D) HBOT+ACNU (combination). Each group had five mice. All treatments were initiated on day 8, when the tumor size reached 40–60 mm^3^. HBO was administrated daily for 3 weeks. ACNU (3 mg/kg; i.p.) was administered once per week for 3 weeks. Tumor size was measured twice per week using a digital caliper, and tumor volume (*V*) was calculated using the formula *V* = [1/2]*ab* ^2^, where *a* and *b* were the long and short diameters of the tumor, respectively. Mice were sacrificed 4 weeks postinjection, following which tumors were carefully removed, and their weight and tumor volume were measured prior to further histological evaluation.

### Hyperbaric oxygen treatment

Mice in the hyperbaric oxygen treatment group were placed into a homemade device for hyperbaric oxygen therapy (specific pathogen‐free level was kept; Fig. 1). HBO was administered at a pressure of 2.5 atm for 90 min. A minimum of 15 min pressurization and depressurization was allowed for the nude mice to adjust to the changes in pressure. The treatment regimen consisted of a 5–10 min ramp‐up to 2.5 atm pressure in a 100% O_2_ environment, followed by sustaining for 90 min at this pressure prior to a 10‐ to 20‐min decompression phase. The hyperbaric oxygen intervention process was performed daily for 21 days.

### Immunohistochemistry

Tumor tissue blocks were cut into 4 *μ*m sections, which were incubated with rabbit polyclonal anti‐HIF1*α* (diluted 1:250), rabbit polyclonal anti‐VEGF (diluted 1:250), rabbit polyclonal anti‐mmp9 (diluted 1:200), rabbit polyclonal anti‐IL‐1*β* (diluted 1:10), rabbit polyclonal anti‐NF‐*κ*B (diluted 1:100), or rabbit polyclonal anti‐TNF‐*α* (diluted 1:150) at 4°C overnight in humid champers. Sections were observed under a laser confocal scanning microscope at a magnification of 400×. Immunohistochemical staining was quantitated using IPP 6.0 image analysis software (Media Cybernetics, USA), and 5–8 fields of view were selected on each section and photographed. Image analyses were performed as described, and mean optical density (MOD) were calculated using the following formula: MOD = Integral optical density/area of interest. MOD was obtained for the various fields of view in each section.

### Western blot analysis

Tumor tissues were cut into pieces, rinsed twice with ice‐cold PBS, and solubilized in lysis buffer containing 20 mmol/L Tris (pH 7.5), 135 mmol/L NaCl, 2 mmol/L EDTA, 2 mmol/L DTT, 25 mmol/L *β*‐glycerophosphate, 2 mmol/L sodium pyrophosphate, 10% glycerol, 1% Triton X‐100, 1 mmol/L sodium orthovanadate, 10 mmol/L NaF, 10 *μ*g/mL aprotinin, 10 *μ*g/mL leupeptin, and 1 mmol/L phenylmethylsulfonyl fluoride (PMSF) for 30 min. Lysates were centrifuged (15,000*g*) at 4°C for 15 min. Equal amounts of the soluble proteins were denatured in SDS, electrophoresed on a 12% SDS‐polyacrylamide gel, and transferred to a polyvinylidene difluoride (PVDF) membrane (Roche Applied Science, USA), and probed with indicated antibodies, respectively. The IgG secondary antibody was used and the blots were developed using TMB immunoblotting system.

### Hematoxylin–eosin staining

Tumor tissues were fixed overnight at 4°C in 4% paraformaldehyde, incubated in 30% sucrose and optical cutting temperature compound (OCT), frozen, and sectioned at 5 *μ*m. Sections were stained with hematoxylin and eosin (H&E) and observed by fluorescence microscope at 200× magnification.

### Ethics statement

Research reported in this manuscript was performed with the approval of the ethics committee of the second affiliated hospital of Soochow University. Animal studies were approved by the Soochow University Animal Care and Use Committee, and they followed internationally recognized guidelines. Animals were euthanized by slow (20%/minute) displacement of chamber air with compressed CO_2_ delivered through a precision flowmeter. The animals were subjected to cervical dislocation as a secondary means to ensure death. All efforts were made to minimize suffering. Details of the animal welfare and steps taken to ameliorate suffering were in accordance with the Regulations for the Administration of Affairs Concerning Experimental Animals approved by the State Council of the People's Republic of China.

### Statistical analysis

All data were expressed as means ± SEM. Statistical analyses were performed by one‐way ANOVA followed by Bonferroni's posttest for multiple comparisons. The statistical analyses were performed using the (SPSS 19.0, USA) software package. *P* < 0.05 was considered to be statistically significant.

## Results

### Survival status, tumor volume, and weight of the tumor‐bearing mice

During the experiment, dietary habits, defecation patterns, and daily activities of the animals were normal, and no accidental deaths occurred. Weight gain or loss in each group was not obvious (Table 1); and the peripheral hemogram related to ACNU treatment induced no corresponding clinical symptoms, although white blood cell count was reduced from pretherapeutic 15.0 × 10^9^/L to posttherapeutic 3.83 × 10^9^/L, which was revealed to be reversible in the following review. According to the tumor length and short diameter measured by a caliper, tumor volume was converted and tumor growth curve was drawn, which demonstrated that the tumor growth rate reduced in the four groups, successively. The starting time of negative acceleration in the proliferation rate reflected by the curve showed an observable initiation in the D group (HBOT+ACNU combination) 16 days after inoculation. In addition, the C group (ACNU treatment) presented an initial negative acceleration in proliferation rate 22 days after inoculation. In contrast, the proliferation rate of the B group (HBOT treatment) and the A group (control) was nearly the same, while the absolute volume and mass were smaller in the B group than those of the A group.

**Table 1:** Evaluation of drug toxicity based on the body weight change

Group	IBW	FBW	FBW/IBW
Control	18.50 ± 0.95	24.17 ± 1.33	1.3
HBOT	18.32 ± 0.60	23.94 ± 2.92	1.31
ACNU	17.94 ± 0.40	20.18 ± 0.93	1.12
HBOT+ACNU	18.07 ± 0.32	19.95 ± 1.62	1.1

The mice were divided into the four groups randomly: control group (untreated), hyperbaric oxygen therapy (HBOT) (only), nimustine (ACNU) (only), and HBOT+ACNU (combination), and each group had five mice, as shown in the Material and Methods section.

**Figure 2:**
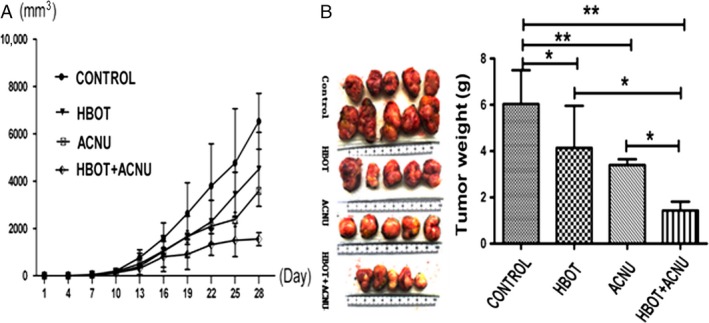
The tumor volume and weight of the tumor‐bearing mice. (A) A comparison of the proliferation rates of transplanted tumors in different groups: proliferation rates in the control group, the hyperbaric oxygen therapy (HBOT) group, the nimustine (ACNU) group, and the HBOT+ACNU group reduced in turn. (B) Comparison of tumor mass in different groups. *P <* *0.05, **P <* *0.01. The mice were divided into the four groups randomly, and each group had five mice, as shown in the Material and Methods section.

At the end of the experiment, the whole tumor tissues were extracted completely, the tumor mass was weighed, and the tumor volume was measured by a caliper, demonstrating that differences in tumor mass, volume, and proliferation curve among four groups showed consistent general trends (Fig. 2A). The tumor mass was 6.03 ± 1.47 g, 4.13 ± 1.82 g, 2.39 ± 0.25 g, and 1.43 ± 0.38 g in A, B, C, and D groups, respectively (*P *<* *0.05, Group B vs. A; *P *<* *0.01, Group C or D vs. A). On the other hand, the tumor weights in D group were significantly smaller than those in both B and C groups (both *P* < 0.05). But it was not statistically significant between B group and C group (*P* > 0.05, Fig. 2B).

We also compared the tumor inhibition rate across the four groups. We found that tumor inhibition rate was 31.51%, 43.79%, and 76.29% in the B, C, and D groups, respectively. We tested if the ACNU and HBOT combination showed synergism based on Jin's formula *Q* = Ea + b/(Ea + Eb – Ea × Eb), where Ea + b, Ea, and Eb are the average effects of the combination treatment, and *Q* < 0.85 indicates antagonism, 0.85 ≤ *Q* < 1.15 indicates additivity, and *Q* ≥ 1.15 indicates synergism 11. Our *Q* value was 1.24, indicating ACNU, and combination treatment showed synergistic effect.

### Tumor tissue pathology and inflammatory cell infiltration analysis

**Figure 3:**
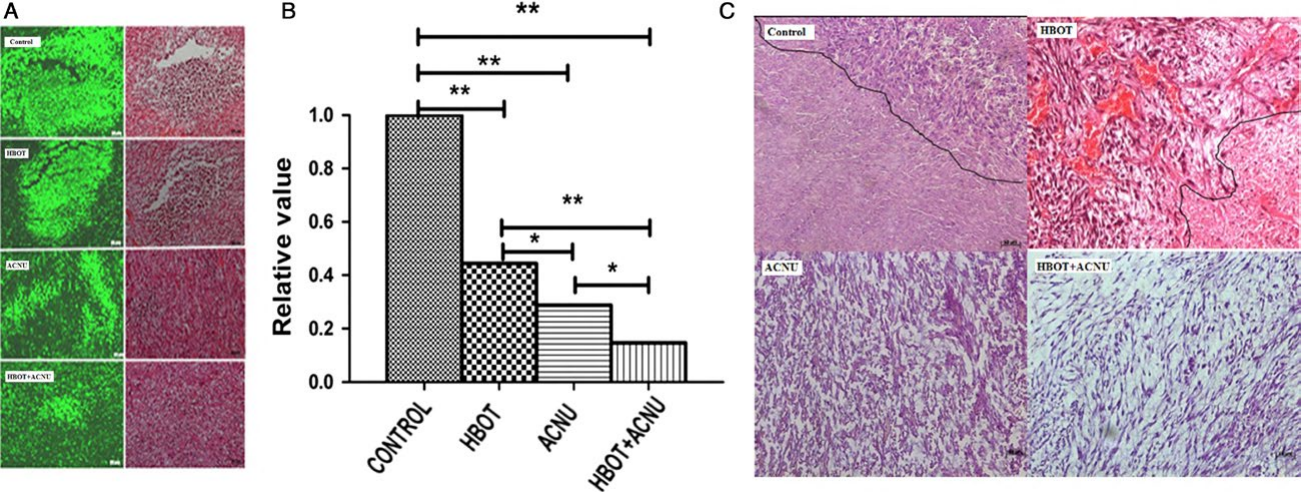
Observation of host cell infiltration (green) in necrotic tumor tissue and H&E‐stained transplanted tumors. (A) Host cell infiltration (green) in necrotic tumor tissue under a microscope. One H&E‐stained tissue section under fluorescent light and white light of a fluorescent microscope. The numbers of infiltrating cells with green fluorescence were different between the groups due to various sizes of necrotic areas; (B) for host cell infiltration (green) in necrotic tumor tissue, the relative ratios of the total intensity of green fluorescence in the control group to those in the other groups measured by Image‐Pro Plus 6.0 software. (C) H&E‐stained transplanted tumors under an optical microscope. The control group: necrotic areas with unclear tissue structure on the bottom left of the figure and non‐necrotic areas on the upper right, with a compact oncocytes arrangement, deeply stained nucleus, and distinct morphological changes. The hyperbaric oxygen therapy (HBOT) group: necrotic areas with unclear tissue structure on the bottom right of the figure and non‐necrotic areas on the upper left, with a compact oncocytes arrangement, deeply stained nucleus, distinct morphological changes, and many blood vessels. It was hard to determine necrotic areas in the nimustine (ACNU) group and the HBOT+ACNU group under an optical microscope. The cell arrangement of non‐necrotic areas was looser in the ACNU group than in the HBOT+ACNU group. The scale bar: 20×. The mice were divided into the four groups randomly, and each group had five mice, as shown in the Material and Methods section. *P < 0.05; **P < 0.01

H&E staining showed that tumors in the control group presented invasive growth, apparent cell heteromorphism, nuclear hyperchromatism, and abundant blood vessels, and the necrosis and hemorrhage were quite common, which were in line with the essential characteristics of the SU3 subcutaneous transplantation tumor, previously reported by our group 12. In contrast, the necrosis and hemorrhage were reduced significantly in the HBOT and ACNU groups, especially in the HBOT+ACNU combined treatment group, which showed no necrotic or hemorrhagic foci basically, loosely arranged cells, significantly reduced interstitial components, and solid tumor. Moreover, in the control group, necrotic foci were detected under a white light microscope, while host‐derived green inflammatory cell infiltration was found under a fluorescence microscope in the same H&E‐stained section, and infiltration area was in accordance with necrotic foci (Fig. 3). Furthermore, total green fluorescence intensity was analyzed, using Image‐Pro Plus6.0 (Silver Spring, USA) medical images. With the A group as basal level (1, 100%), the ratios of the groups B, C, and D were 0.44 (*P *<* *0.05), 0.29 (*P *<* *0.05), and 0.15 (*P *<* *0.05), respectively. These results demonstrated that the degree of necrosis was reduced, and inflammatory cell infiltration also decreased after both HBOT and ACNU treatments. Additionally, these clinical phenotypes were consistent with the changes in tumor volume and tumor mass, with the D group presenting the most obvious phenotypes and changes.

### Molecular regulatory molecules analysis

Since inflammation plays an important role in tumor development, which mainly acts through the medium of a number of inflammatory cytokines, such as IL‐1*β* and TNF‐*α*. Abnormal expression of these factors as well as other carcinogenic factors, such as NF‐*κ*B and MMP9, further promotes tumor progression. Therefore, we further investigated the affect of hyperbaric oxygen on the expression of IL‐1, TNF*α*, and other factors including HIF‐1*α*, NF‐*κ*B, VEGF, and MMP9, which were closely related to tumor development.

**Figure 4:**
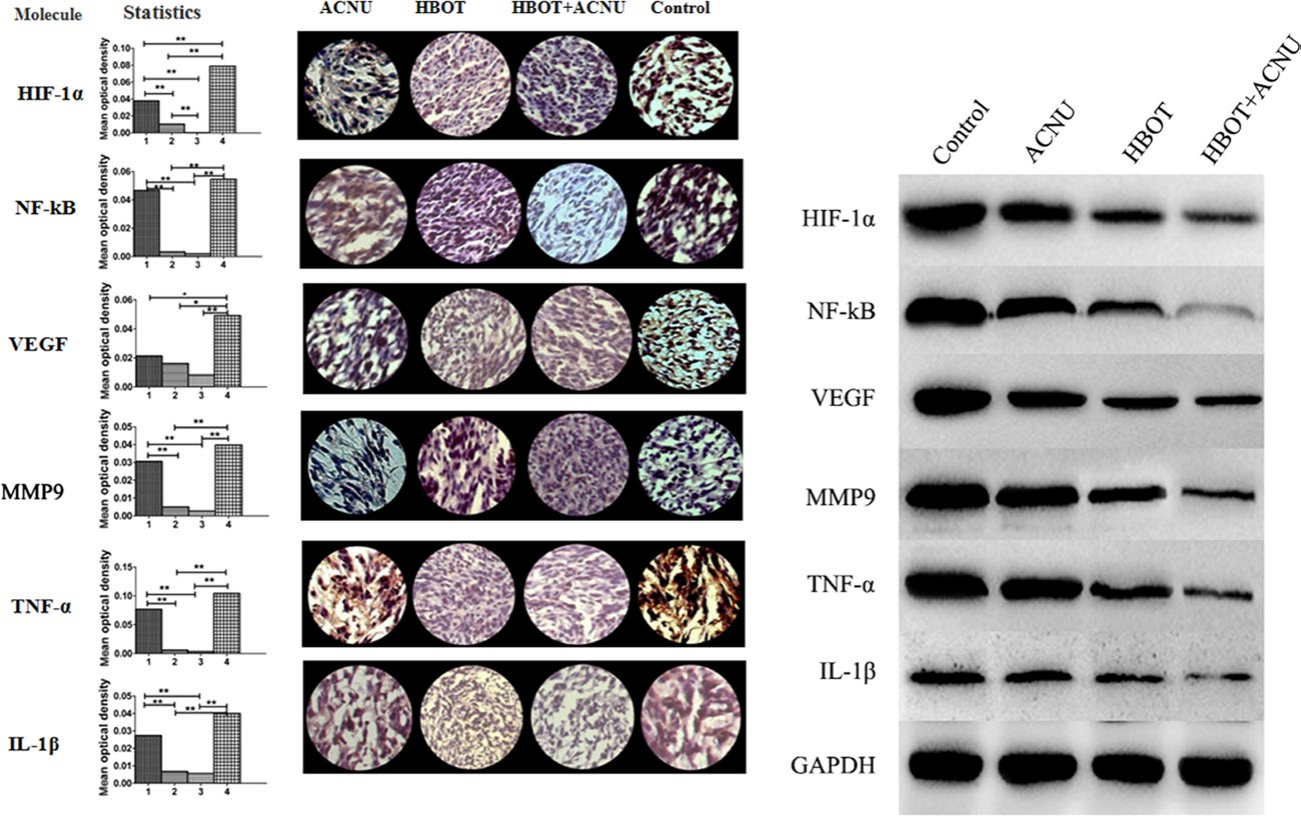
Statistical chart of the gray values obtained from immunohistochemical analysis of relevant proteins in hyperbaric oxygen therapy (HBOT) combined with nimustine (ACNU) therapy. The left side showed the gray values; the middle part showed immunohistochemically stained samples under a microscope (40×); the right side showed expression levels of HIF‐1*α*, TNF‐*α*, IL‐1*β*, VEGF, MMP9, and NF‐*κ*B, using western blotting method, with endogenous GAPDH as internal control. The *x*‐axis of the histogram showed the ACNU group (1), the HBOT group (2), the HBOT+ACNU group (3), and the control group (4), respectively; the *y*‐axis indicated mean optical density. The mice were divided into the four groups randomly, and each group had five mice, as shown in the Material and Methods section. *P < 0.05; **P < 0.01

**Figure 5:**
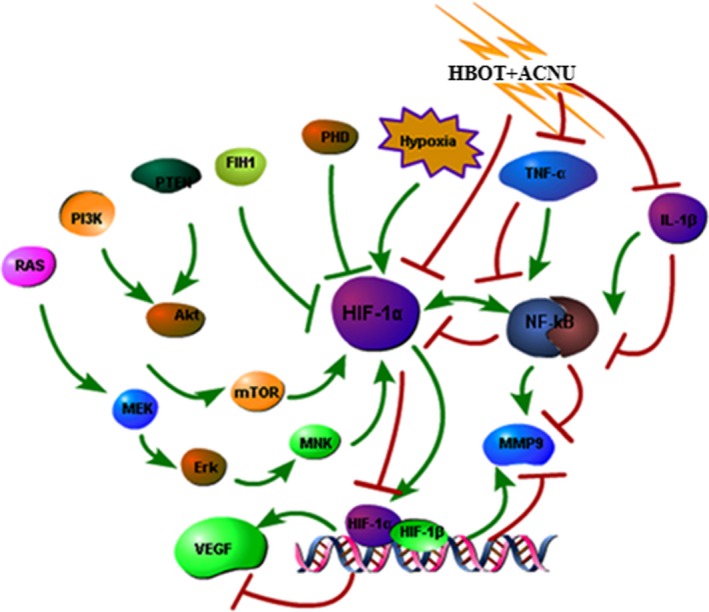
Diagram of hyperbaric oxygen therapy‐induced HIF–TNF–NF‐*κ*B regulatory network.

The expression of the molecules, including TNF‐*α*, IL‐1*β*, HIF‐1*α*, NF‐*κ*B, VEGF, and MMP9 in tumor tissues was analyzed by the methods of immunohistochemistry and western blot. The results of sections were shown in Table 2 and Figure 4. The quantity of all six molecules in tumor tissues from HBOT+ACNU (combination) group and HBOT (only) group were efficiently attenuated, compared with that of control (untreated) group and ACNU (only) group, suggesting HBOT could effectively inhibit the expression of six molecules. The HBOT–ACNU treatment induced molecular regulation network combined with KEGG database data, using Pathway Builder software, as were illustrated in Figure 5, showing that HBO increased the sensitivity of ACNU against glioma by regulating HIF–TNF–NF‐*κ*B signaling pathway.

**Table 2:** Comparison of associated molecular protein in different groups (mean optical density)

Group	HIF‐1*α*	NF‐*κ*B	VEGF	MMP9	TNF‐*α*	IL‐1*β*
ACNU	0.067 ± 0.012	0.046 ± 0.006	0.021 ± 0.004	0.031 ± 0.006	0.076 ± 0.006	0.027 ± 0.001
HBOT	0.016 ± 0.005	0.003 ± 0.001	0.016 ± 0.008	0.005 ± 0.003	0.005 ± 0.003	0.006 ± 0.003
HBOT+ACNU	0.011 ± 0.005	0.001 ± 0.001	0.008 ± 0.003	0.003 ± 0.002	0.003 ± 0.001	0.003 ± 0.005
Control	0.069 ± 0.014	0.05 ± 0.014	0.049 ± 0.018	0.039 ± 0.001	0.0104 ± 0.02	0.03 ± 0.007

The mice were divided into the four groups randomly: control group (untreated), HBOT (only), nimustine (ACNU) (only), HBOT+ACNU (combination), and each group had five mice, as shown in the Material and Methods section.

## Discussion

### Tumor tissue hypoxia and HBOT

**Figure 6:**
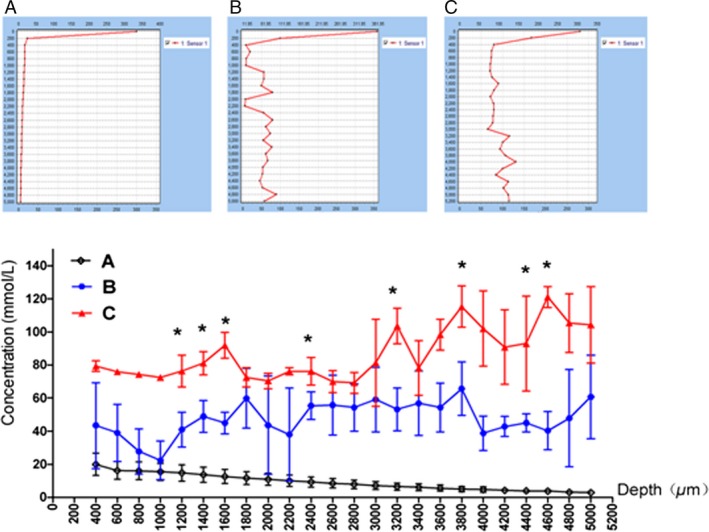
Determination of oxygen content in brain of rats after hyperbaric oxygen therapy (HBOT) by microelectrode. The vertical coordinate was oxygen concentration (mmol/L) and the horizontal coordinate was the probe into the depth of brain tissue (mm).*P <* *0.05. The mice were divided into the four groups randomly, and each group had five mice, as shown in the Material and Methods section.

The presence of tumor tissue hypoxia had become an unquestioned fact. It was found that hypoxia of high‐grade glioma was severe than that of low‐grade glioma, and hypoxia could promote rather than inhibit tumor growth 13, 14, 15. Our team had proved that glioma stem cells could transform into tumor vascular endothelial cell under low PO_2_ condition, and one of the reasons for low PO_2_‐promoting tumor growth was that reconstructed tumor vessels directly participated in tumor blood circulation 16, 17. If HBOT truly enhanced PO_2_ in tumor tissues, then the above‐mentioned debate on HBOT of malignant tumor might arrive at the same conclusion. Therefore, PO_2_ in tumor tissues must be monitored during HBOT experiments on tumor‐bearing animals, to avoid the situation, in which the tumor tissue PO_2_ was not enhanced owing to some reasons from HBOT itself. Otherwise, the discussion on the efficacy of HBOT would lack the most basic evidence. Kayama et al. 18 directly measured oxygen pressure (PO_2_) in tumor tissue, which was significantly lower than that in surrounding brain tissue. We determined PO_2_ in the model of this experiment with unisense microelectrode, showing that PO_2_ in the brain of mice treated with HBOT, was much higher than that in normal mouse, and that tumor tissue PO_2_ was also enhanced and was getting close to that in normal mouse brain (Fig. 6).

### Molecular regulatory pathways influenced by HBOT

We had previously reported that reversible malignant cell proliferation and necrotic tumor tissue in the present tumor model were related to high expression of HIF‐1*α*, TNF‐*α*, CD68, and CD11b 16, 17. The control group in this study was a copy of the model, with not only HIF‐1*α* and TNF‐*α*, but also IL‐1*β*, VEGF, MMP9, and NF‐*κ*B being highly expressed. However, their expression was reduced by HBOT to inhibit tumor proliferation. This had become a molecular evidence for effectiveness of HBOT on glioma and sensitizing effect of HBOT on ACNU, but its regulatory pathway still needed to be illuminated. Together with the data from KEEG database, we believed that the above six molecules basically covered the sources of tumor and inflammatory cell, while nonresolving inflammation was the major cause of almost all malignant tumors 19. Highly expressed inflammatory factors such as IL‐1*β* and TNF‐*α*, markers of inflammatory cells such as CD68 and CD11b, and massive inflammatory cells tracked by fluorescence in necrotic tumor tissue were detected in the tumor tissue of this model (Fig. 3). It suggested that this model was a copy of glioma and the genesis and progression of glioma were closely connected to nonresolving inflammation. Hypoxia‐inducible factor (HIF) reported by Kumar and Gabrilovich 20 was one of the biggest concerns, as it was upregulated in cell responses to low PO_2_, and this promoted immunosuppressive activity of nonresolving inflammatory cells such as MDSC (myeloid‐derived suppressor cells) and TAM (tumor‐associated macrophages) and accelerated transformation from MDSC into TAM.

TNF*α*, IL‐1*β*, and NF‐*κ*B are also related to nonresolving inflammation. TNF includes TNF‐*α* and *β*, which are generated by macrophage and lymphocyte, respectively. As a double‐edged sword, TNF not only could induce tumor cell apoptosis 21 and enhance immune function 22, but also could induce expression of VEGF, MMPs, and sVCAM‐1, and promote angiogenesis and tumor cell metastasis 23, 24. IL‐1 mainly includes IL‐1*α* and IL‐1*β*. IL‐1*β* is widely expressed. Besides macrophages and monocytes, it had been reported by Tarassishin et al. 25 that glioma cells also had high expression of IL‐1*β*. Immunological homeostasis maintains an absent or low expression of IL‐1*β*, which could only be upregulated by stimulation of inflammatory signals 26, 27. Large‐dose IL‐1*β* promotes expression of IGF/HGF, MMPs, MCAM, VEGF, and NLRP3, and played a role in tumor angiogenesis, cell invasion, and dispersion 25, 28, 29, 30.

In conclusion, HBOT could inhibit glioma cell proliferation and inflammatory cell infiltration, and also exerted a sensitizing effect on ACNU therapy. This was associated with the fact that HBOT enhanced PO_2_ in tumor tissues and thus leaded to low expression of HIF‐1*α*, TNF‐*α*, IL‐1*β*, VEGF, MMP9, and NF‐*κ*B. Therefore, hyperbaric oxygen therapy might be a potentially effective therapeutic option and might improve the prognosis for patients with glioma. The combination of hyperbaric oxygen with ACNU therapy produced an important reduction in glioma growth and effective approach to the treatment of glioblastoma.

## Conflict of Interest

The authors declare no conflict of interest.
